# Identification of cytokine-induced modulation of microRNA expression and secretion as measured by a novel microRNA specific qPCR assay

**DOI:** 10.1038/srep11590

**Published:** 2015-06-25

**Authors:** Vladimir Benes, Paul Collier, Claus Kordes, Jens Stolte, Tobias Rausch, Martina U. Muckentaler, Dieter Häussinger, Mirco Castoldi

**Affiliations:** 1 European Molecular Biology Laboratory, Genomics Core Facility, D 69117 Heidelberg, Germany; 2 Department of Gastroenterology, Hepatology and Infectious Diseases, Heinrich Heine University, D 40225 Düsseldorf, Germany; 3 RIKEN 1 230 0045, 1 Chome-7-22 Suehirochō, Tsurumi-ku, Yokohama-shi, Kanagawa-ken 230-0045, Japan; 4 Department of Pediatric Oncology, Hematology and Immunology University of Heidelberg, D 69120 Heidelberg, Germany; 5 Molecular Medicine Partnership Unit, University of Heidelberg, D 69120 Heidelberg, Germany

## Abstract

microRNAs are an abundant class of small non-coding RNAs that control gene expression post-transcriptionally. Importantly, microRNA activity participates in the regulation of cellular processes and is a potentially valuable source of biomarkers in the diagnosis and prognosis of human diseases. Here we introduce miQPCR, an innovative method to quantify microRNAs expression by using Real-Time PCR. miQPCR exploits T4 RNA ligase activities to extend uniformly microRNAs’ 3′-ends by addition of a linker-adapter. The adapter is then used as ‘anchor’ to prime cDNA synthesis and throughout qPCR to amplify specifically target amplicons. miQPCR is an open, adaptable and cost-effective procedure, which offers the following advantages; i) universal elongation and reverse transcription of all microRNAs; ii) *Tm*-adjustment of microRNA-specific primers; iii) high sensitivity and specificity in discriminating among closely related sequences and; iv) suitable for the analysis of cellular and cell-free circulating microRNAs. Analysis of cellular and cell-free circulating microRNAs secreted by rat primary hepatocytes stimulated with cytokines and growth factors identifies for the first time a widespread modulation of both microRNAs expression and secretion. Altogether, our findings suggest that the pleiotropic activity of humoral factors on microRNAs may extensively affect liver function in response to injury and regeneration.

microRNAs (miRNAs) are short, non-coding RNAs (ncRNAs) that control gene expression at the post-transcriptional level^1^. miRNAs are transcribed within long primary transcripts that are processed via two successive RNase III-like enzyme mediated-cleavage steps. Drosha conduces the first processing step in the nucleus by cleaving the primary transcripts to produce a double-stranded precursor (2; 80–100 nts long). The second processing step occurs in the cytoplasm where Dicer generates a short duplex RNA, consisting of a guide and passenger strands (3; 19–24 nts long). Although either strand of the duplex may act as a functional miRNA, the carrier strand is preferentially incorporated into the RNA-induced silencing complex (4; RISC) where, in a sequence specific fashion, interacts with the 3′-untranslated regions of target mRNAs either impairing mRNA translation or stability^5^. Recent studies demonstrated the intimate connection between miRNA expression and regulation of development^6^,^7^, differentiation^8^,^9^ and metabolism^10^,^11^. Furthermore, considering that aberrant miRNA expression has been linked to the pathogenesis of human diseases^12^,^13^,^14^, changes in the expression of specific miRNAs may potentially provide valuable diagnostic and prognostic information^15^. In addition to cellular miRNAs, cell-free circulating miRNAs have been isolated in body fluids^16^ and detected in tissue culture medium^17^. To date, two different populations of cell-free circulating miRNAs have been identified; one included in exosomes and one associated to the Argonaute proteins^18^. To date no function has been found for the Argonate-associate miRNAs. However, exosomal-miRNAs are heavily investigated as potential source of disease-associated biomarkers. Exosomes, are nano-sized (30–200 nm) transporters involved in cell-to-cell communication through the shuttling of proteins, RNAs, and lipids between cells^19^. The existence of a specific population of exosomal-associated miRNAs circulating in the blood of healthy as well as diseased individuals have raised the possibility that analysis of these miRNAs may identify clinically relevant biomarkers for the early detection and in the monitoring of human diseases^20^.

As miRNAs are isolated from heterogeneous sources with different methodologies, it is important to have reliable, reproducible and robust tools for the quantification of miRNA expression by using qPCR. Importantly, the method of choice should convey the different experimental set-ups used to isolate miRNAs (i.e. cellular vs exosomal) into a unique down-stream application. To date various commercial methods dedicated to miRNA detection by qPCR are available. However, commercial platforms are not particularly flexible to user needs, nor readily adaptable to the increasing numbers of known miRNAs. Additionally, commercial platforms frequently only provide miRNA-specific primers covering primarily human, mouse and rat as model organisms. Therefore, researchers working with ‘non-canonical’ animal models or seeking to validate newly discovered miRNAs do not have ready access to this technique. In order to overcome these limitations, we have developed an ‘open source’ miRNA specific qPCR platform named ‘miQPCR’. The miQPCR approach is based on well-established techniques as it exploits the activity of truncated T4 RNA ligase 2 (Rnl2tr) to join the 5′-end of a linker adaptor to the 3′-end of single-stranded RNAs (21,22; ssRNAs) including miRNAs. Standard approaches are used to reverse transcribe linker-tagged miRNAs and a fraction of the synthesized cDNA is amplified and quantified in a qPCR assay.

Several published studies indicate that miRNAs and cytokine activities are strongly interconnected, as it was shown that miRNAs expression is modulated in response to cytokine stimulation^23^, while cytokine expression is regulated by miRNAs^24^,^25^. As miRNAs are pivotal in the modulation of liver function^26^, it is expected that cytokines or growth factors will modulate function of hepatic miRNAs during inflammation or liver injury and regeneration. However, to the best of our knowledge the modulation of miRNA activity by cytokines or growth factors in the liver has not been systematically investigated. In this study we employed miQPCR to analyze the activity of growth factors [Fibroblast Growth Factor 2 (basic); FGF2, Fibroblast Growth Factor 4; FGF4 and Hepatocyte growth factor; HGF] and cytokines (Interleukin-1 alpha; IL-1α, Interleukin-6; IL-6, Interferon-beta; INF-β, and Transforming Growth Factor-beta1; TGF-β1) on both, the expression and secretion of a panel of selected miRNAs in cultured rat primary hepatocytes. To the best of our knowledge, this is the first study showing that cytokines and growth factors are able to modulate both the expression and the secretion of miRNAs in cultured primary hepatocytes. Importantly, we identified that FGF2, FGF4 and INF-β, down-regulated the expression of several miRNAs in the cultured hepatocytes, while IL-6, INF-β and TGF-β1 are able to modulate the quantity of exosomal-miRNAs secreted by the primary hepatocytes into the culture medium. Altogether, our data indicate that the pleiotropic effect of cytokines and growth factors on miRNAs expression and secretion might have an extensive effect on liver function whilst the liver is recovering from different insults or during chronic and acute liver diseases.

## Results

### miQPCR workflow, miRNA elongation and reverse transcription

**Figure 1:**
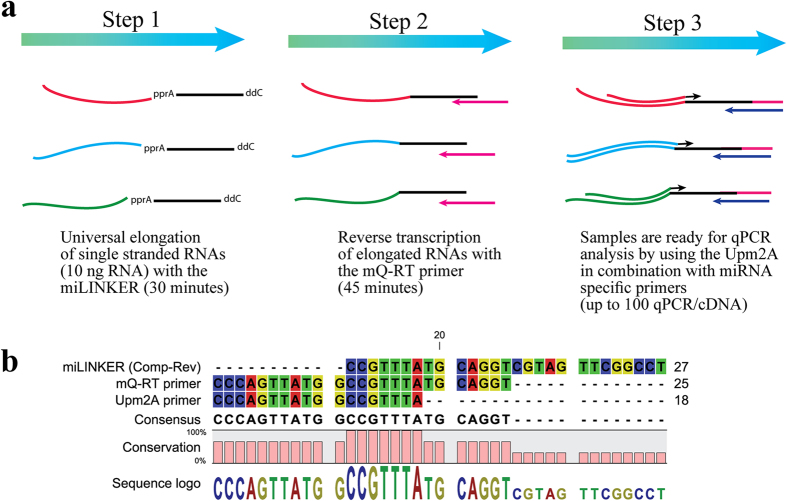
Workflow of cDNA synthesis following the miQPCR approach. **a**) During the first step the 3′-end of ssRNA is enzymatically ligated to the 5′ end of miLINKER by truncated T4 RNA ligase (time ~30 min). In the second step, a reverse transcriptase is used to reverse transcribe elongate miRNAs into cDNA with a universal reverse-transcription primer (mQ-RT, time ~45 min). In the third and final step, cDNA is diluted, the miRNA of interest is amplified by qPCR using a reverse universal primer (Upm2A) and a forward miRNA-specific primer while the amplicon is detected by using SYBR Green I. **b**) Multiple alignment among the complement reverse of the linker adaptor (miLINKER) and the sequences of the reverse transcription primer (mQ-RT) and of the universal qPCR primer (Upm2A). All the sequences are indicated in the 5′ → 3′ orientation. For miLINKER, mQ-RT and Upm2A sequences see Supplementary Table 1a and 1b.

miRNA expression profiling is challenging as mature miRNAs are; i) short single stranded RNAs (22–24 nts); ii) their CG content varies between 33% of hsa-miR-144 and 89% of hsa-miR-4665-3p resulting in a wide range of *Tms*; iii) miRNAs only represent a small fraction of the cellular RNA; iv) miRNA target sequence is contained in its precursors (i.e. pri- and pre-miRNAs); and v) miRNAs are redundant and exist in families where individual members can differ by just a single nucleotide^27^. To overcome these limitations, we have developed an innovative approach for cDNA synthesis, where miRNAs’ 3′-ends are universally elongated via the ligation of a 26 nts long oligonucleotide adaptor (named miLINKER). Thereafter, the reverse transcription of elongated miRNAs is primed by the annealing to an optimized reverse transcription primer (named mQ-RT). An important characteristic of the presented method is that the miLINKER does not contain the full sequence for the universal qPCR primer (named Upm2A), as the missing portion of the sequence is introduced in the amplicon during the reverse transcription via the mQ-RT primer. Reasons for this include an increased specificity of the qPCR assay as well as in the possibility of modifying or adapting the universal qPCR primer without the need of changing the miLINKER sequence. The complete procedure, which requires 10 ng of total RNA, is generally carried out in 75 minutes and up to 100 individual qPCR assays can be carried out from a single cDNA (Fig. 1a; see Table 1 and on-line Material and Methods for step-by-step miQPCR protocol, optimized qPCR master mix calculator and qPCR cycler program).

### miLINKER design

To elongate miRNAs uniformly and to perform cDNA synthesis, miQPCR relies on the ligation of an adenylated linker adaptor (the miLINKER), to miRNA 3′-ends^28^. Significantly, as this reaction is conducted in absence of ATP the formation of circularized RNAs as a ligation byproduct is greatly reduced^22^. The miLINKER is a 26 nts long DNA oligo where the 5′-end present a 5′, 5′-adenyl group, while a Dideoxycytidine (ddC) blocks the 3′-end of the miLINKER. Notably, the miLINKER sequence was derived from the Solanum tuberosum (*potato*) phyB gene (29; PHYB). PHYB gene, which is species-specific, encodes for a photoreceptor involved in the development of purple coloration in potato root. Due to its unique features, PHYB has been previously used for developing the ‘SPUD assay’, which was created to assess the presence of polymerase inhibitors in qPCR assays^30^.

### Design and optimization of miRNA-specific primers

**Figure 2:**
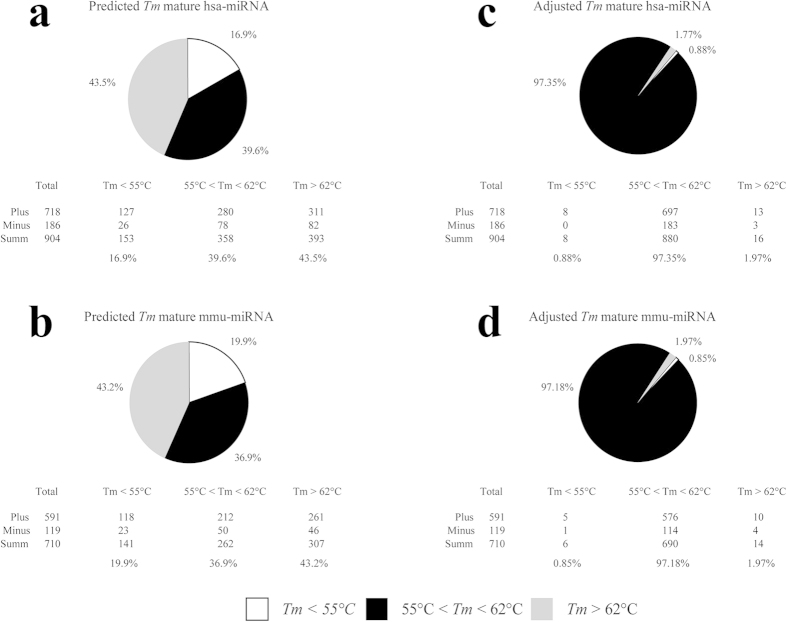
*Tm* prediction of human and mouse miRNAs contained in the miRBase v19. Analysis of the melting temperature (*Tm*) indicate that around 40% of the human **a**) and 37% of the mouse **b**) sequences show a predicted *Tm* within the optimal range for qPCR amplification (55 °C < *Tm* < 62 °C). More than 43% of the sequences (for both human and mouse) show a predicted *Tm* above the optimal range (*Tm* > 62 °C) while less than 20% displayed a predicted *Tm* below the optimal range (*Tm* < 55 °C). By implementing miQPCR primer design (For complete prediction see Supplementary Table 2) we were able to adjust the predicted *Tm* for most of the primers (~97%, **c** human and **d** mouse) to fall within the optimal conditions for qPCR assays (55 °C < *Tm* < 62 °C).

The specificity and sensitivity of qPCR assays rely on primer design. For the intrinsic nature of the mature miRNAs, the design of miRNA-specific primers is particularly challenging. miRNAs have highly heterogeneous GC content resulting in a wide range of predicted *Tm*. As qPCR assays are run at 60 °C, differences in annealing temperatures are likely to impair either the specificity (predicted *Tm* > 62 °C) or sensitivity (predicted *Tm* < 55 °C) of a number of miRNA assays. To evaluate the *Tm* distribution across the miRNAs encoded in mammalian genomes, *Tm* prediction for all the human- and mouse-miRNAs included in the miRBase [version 19;^27^] was carried out (Fig. 2). This approach indicates that ~40% of the human and mouse miRNAs had predicted *Tms* within what we consider the optimal qPCR range (i.e. between 55 °C and 62 °C). Whereas the remnant miRNAs predicted *Tms* were either above (~40% miRNAs *Tm* > 62 °C) or below (~20% miRNAs *Tm* < 55 °C) the optimal qPCR range (Fig. 2a,b). A major advantage of the miQPCR method is that following elongation and reverse transcription the length of the target amplicon is around 60 nts instead of 22–24 nts. Hence, the sequence of miRNA-specific primers can be adjusted to achieve a consistent *Tm* across different assays. This is (empirically) achieved through either the shortening of the 5′-ends of primers with a predicted *Tm* higher than 62 °C or elongating the 3′-ends of primers with a predicted *Tm* lower the 55 °C into the miLINKER’s 5′-end. As a proof of principle, this empirical method was applied in the design of gene specific primers targeting all human and mouse miRNAs contained in the miRBase (version 19; Fig. 2c,d). *Tm* prediction carried out on human and mouse optimized primers shows that the *Tms* of ~96% of miQPCR-designed primers falls within the desired *Tm* range (see on-line Material and Methods for the stepwise optimization of miRNA primer design. The sequences of *Tm* adjusted primers of all miRNAs contained in miRBase version 19 are available in the Supplementary Table 2).

### Specificity and sensitivity assessment of miQPCR assays

**Figure 3:**
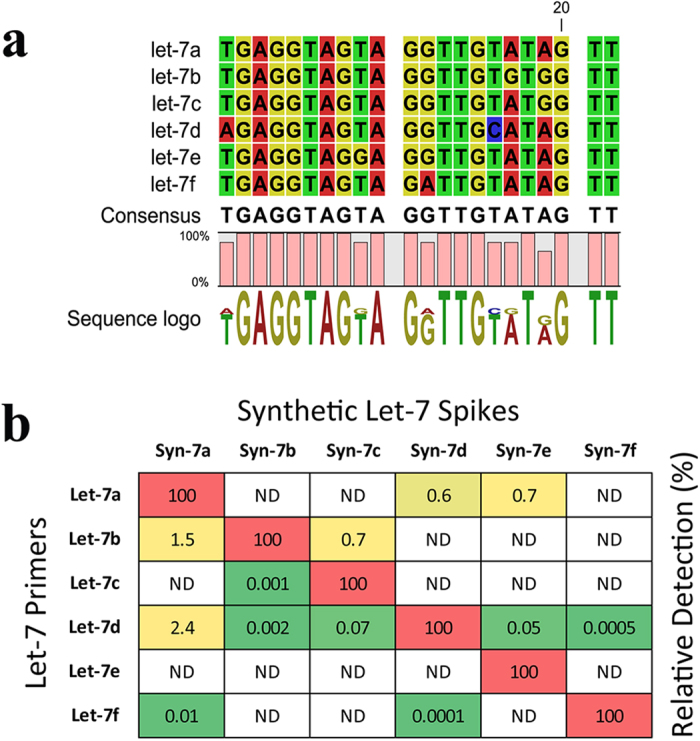
miQPCR assay discriminates among the closely related members of the Let-7 family. **a**) Multiple alignment of the six selected members of the Let-7 family. **b**) Six members of the Let-7 family (Let-7a, Let-7b, Let-7c, Let-7d, Let-7e and Let-7f) were individually spiked into yeast total RNA. Following, 10 ng of yeast total RNA containing 2*10^8^ copies of the selected miRNA (or 3.3 fmol) were reverse transcribed according to the miQPCR protocol and 100 pg of yeast RNA (containing 2*10^6^ copies of each individual miRNA) were analyzed for cross reactivity. Values in the panel B represent the relative detection (expressed in percentage) calculated based on Ct difference between the perfectly matched and mismatched targets. Data were calculated from six independent cDNAs synthesis.

To assess miQPCR specificity in discriminating between closely related sequences we focused on the analysis of the Let-7 family. The analysis of these miRNAs poses several challenges, as members of this family are nearly identical (Fig. 3a). To test specificity, separate assays were designed to monitor the cross reactivity of six related members of the Let-7 family (Let-7a, Let-7b, Let-7c, Let-7d, Let-7e and Let-7f). As shown in Fig. 3b every assay tested exhibited substantial target sequence specificity. Importantly cross talk between primers designed to amplify specifically a member of the family and other members of the Let-7 family was marginal and with limited overlap. Overall, these data indicate that miQPCR assay is able to discriminate among closely related sequences.

**Figure 4:**
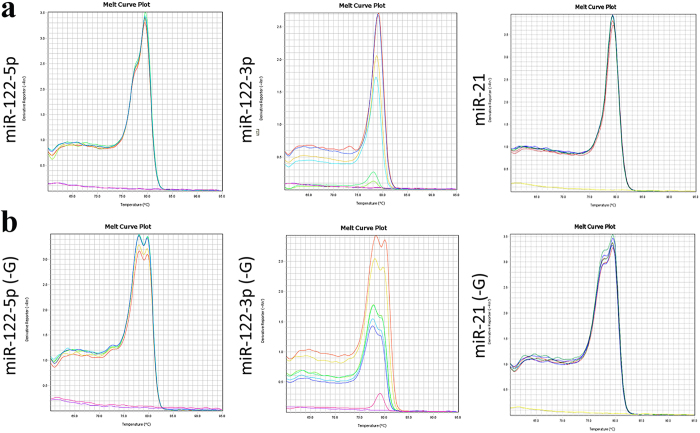
Discrimination between mature miRNAs and their precursors. To identify whether miQPCR primer design is able to discriminate between mature miRNAs and their precursors miRNA-specific primers targeting miR-122-5p, miR-122-3p and miR-21-5p were designed according to the standard miQPCR design protocol (i.e. containing a 3′ end ‘G’ overlapping with the miLINKER) or without miLINKER overlap. **a**) The primers designed with miLINKER overlap produces single pick melting curves, while **b**) the amplification products generated by the ‘G-less’ primers have melting curves with double picks. Melting curves from six biological replicas are shown. Negative RT and NTC did not show any amplification.

To the best of our knowledge, the truncated T4 RNA used in the miQPCR protocol (i.e. Rnl2tr) will elongate all the RNAs presenting an accessible single stranded 3′ end, including many miRNA-precursors. However, the primer design is what ensures the discrimination between the mature and precursor miRNAs. As proof of principle, miRNA-specific primers targeting miR-122-5p, miR-122-3p and miR-21-5p were designed following the standard miQPCR design and without the additional 3′ end ‘G’ (for sequences see Supplementary Table 1a). Importantly, standard miQPCR primers generates a single specific melting curve (Fig. 4a), while the ‘G-less’ primers generated melting curves with two distinct peaks (Fig. 4a). Overall, these data show that the miQPCR assay enables the design of miRNA specific primers discriminating between mature miRNAs and their precursors.

**Figure 5:**
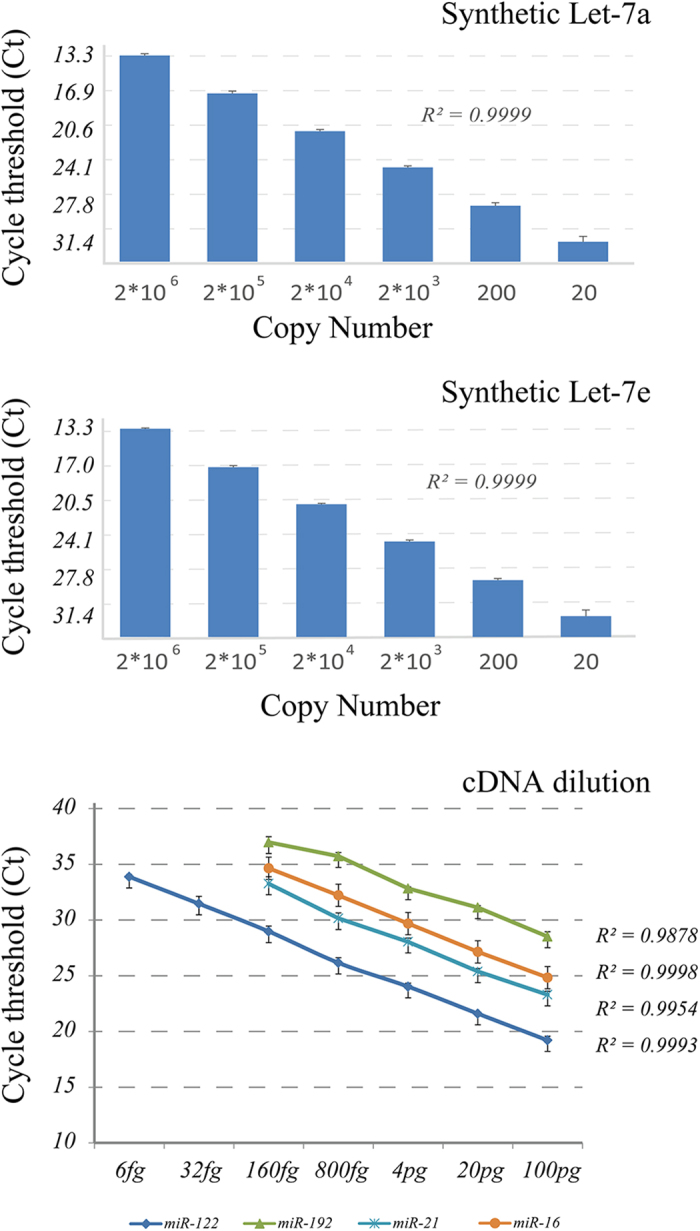
miQPCR approach displays a wide dynamic range and high sensitivity. Top and middle panels; Two members of the Let-7 family (Let-7a and Let-7e) were spiked into yeast total RNA and 10 ng of yeast total RNA containing 2*10^8^ copies of the selected miRNA (or 3.3 fmol) were reversed transcribed using the miQPCR. Following cDNA synthesis 100 pg of yeast RNA (containing 2*10^6^ copies) were used to create five 1:10 linear dilution, which were analyzed by qPCR. Data are represented as average ± standard deviation calculated from six independent cDNAs synthesis. Analysis indicates that miQPCR can detect as little as 20 copy of the target miRNAs and that the detection of the analyzed targets sequence is linear (as shown by the linear regression R^2^). Lower panel; To evaluate the performance of the miQPCR in a physiological context, liver total RNA was reverse transcribed and 100 pg of cDNA were used to prepare 1:5 scalar dilutions (100 pg, 20 pg, 4 pg, 800 fg, 160 fg, 32 fg and 6 fg). Next, the expression of four endogenous miRNAs (miR-122, miR-192, miR-21 and miR-16) was analyzed by qPCR, showing that the detection of the analyzed targets sequence is linear (as shown by the linear regression R^2^). For highly abundant RNA targets (i.e. miR-122), the detection by qPCR maintains its linearity also when the input material is greatly diluted. Data are represented as Ct average ± standard deviation calculated from four independent cDNAs synthesis.

The dynamic range and sensitivity of the miQPCR assays were evaluated using synthetic Let-7a and Let-7e (Fig. 5). Copy numbers of synthetic miRNAs were calculated based on their concentration and cDNAs were diluted over six orders of magnitude (Fig. 5a,b). The miQPCR assays displayed linearity between the input copy number of target and Ct value, and it is capable of detecting as little as twenty copies in the PCR assay. To further evaluate miQPCR dynamic range extensively, four additional miRNA assays (miR-122, miR-192, miR-21 and miR-16) were analyzed in cDNA synthesized from mouse liver RNA (Fig. 5c and in Supplementary Figure 5). Analysis of standard curves shows that endogenous miRNAs are detected over several orders of magnitude, while abundant miRNAs such as the liver specific miR-122^31^ are detected from as little as 6 fg of cDNA. To summarize, the presented data demonstrate that the cDNA synthesis following the miQPCR protocol enables for the design of highly sensitive and specific primers, which efficiently discriminate between closely related sequences.

### miQPCR comparison with established microRNA profiling technologies

**Figure 6:**
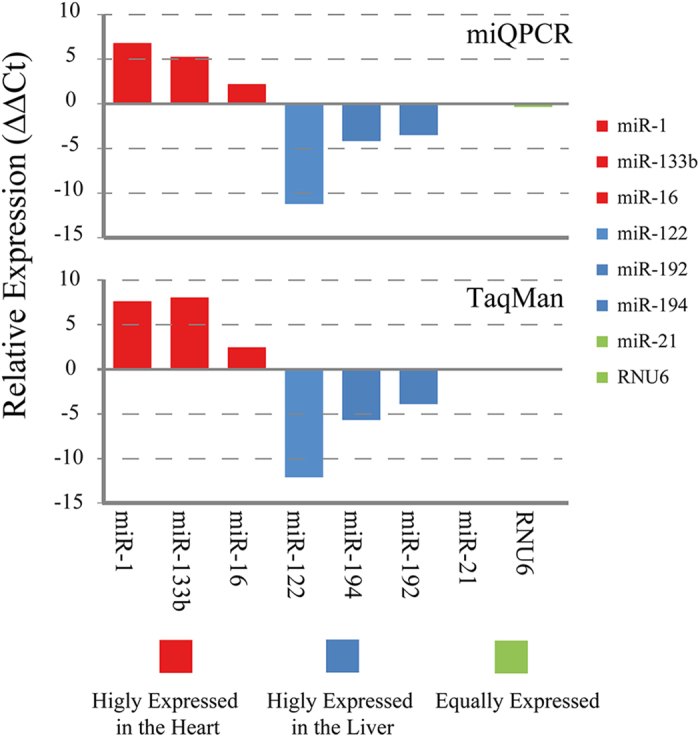
Cross platform comparison of miRNA expression between TaqMan and miQPCR miRNA assays. Total RNA from heart or liver were reverse transcribed according to the miQPCR and TaqMan protocols and the expression of 7 miRNAs (miR-1, miR-133b, miR-16, miR-122, miR-194 and miR-21) and a small nuclear RNA (RNU6) was measured by qPCR with respectively SYBR-Green (top panel) or TaqMan probes (lower panel). Data shows a nearly perfect correlation between the TaqMan and the miQPCR platform. For each assay, Cts from four independent cDNAs synthesis were averaged and the ΔΔCt of heart vs liver were calculated. For actual Cts values (represented as average ± standard deviation) and for the comparison with microarray data see Supplementary Figure 1.

To assess whether miQPCR delivers data comparable to commercial miRNA-qPCR platforms, we benchmarked miQPCR against the ‘gold-standard’ for quantification of miRNA expression by qPCR, the TaqMan miRNA assays^32^. For this purpose, FirstChoice total RNAs (Life Technologies) isolated from either mouse liver or heart were labeled and hybridized to the miCHIP microarray platform as previously described^33^. Microarray analysis identified a panel of miRNAs, which are either highly expressed in the heart (miR-1, miR-133a and miR-16) or in the liver (miR-122, miR-192 and miR-194) or invariant (miR-21; Supplementary Figure 1a). The expression of the selected miRNAs and RNU6 was measured by using either TaqMan or miQPCR approaches (Fig. 6 and Supplementary Figure 1b). The data show a perfect overlap between the two qPCR platforms, showing that the miRNA profiling by using miQPCR (Fig. 6, higher panel) and TaqMan (Fig. 6, lower panel) accurately reflect the miRNA expression patterns in the mouse liver and heart as measured by microarrays. Importantly, cDNA synthesis and analysis by miQPCR compared to the TaqMan approach required less material (i.e. less RNA), reduced workload (i.e. less pipetting) and considerably reduced the costs per qPCR assay.

### Cytokines and growth factors modulate both the expression and the secretion of miRNA in cultured rat primary hepatocytes

**Figure 7:**
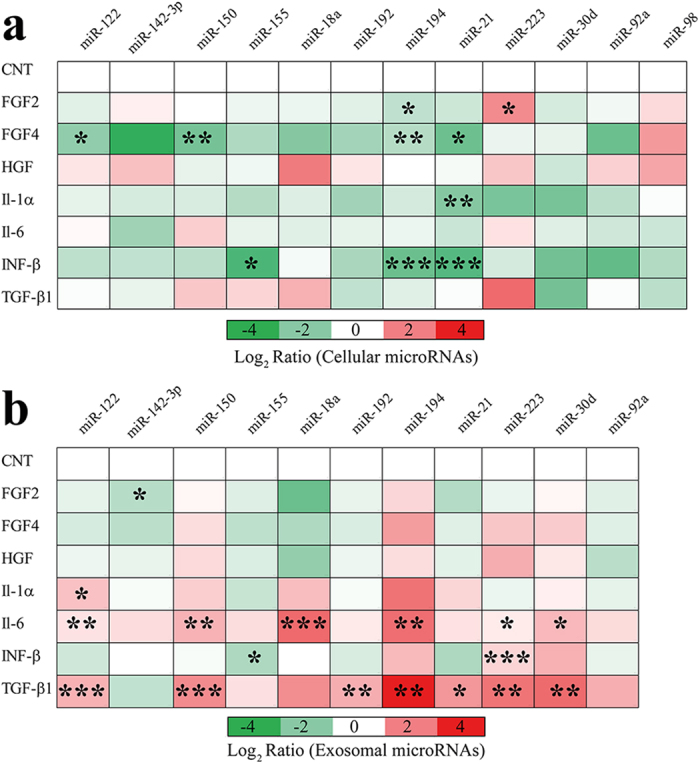
Modulation of miRNA expression and secretion in rat primary hepatocytes stimulated with cytokines and growth factors. Rat primary hepatocytes were stimulated with growth factors (FGF2, FGF4 and HGF) or cytokines (IL-1α, IL-6, INF-β and TGF-β1) for 24 hours. Expression profiling of a selected panel of miRNAs was analyzed by using miQPCR in either cellular **a**) or exosomal **b**) RNAs. Statistical analysis was performed by unpaired T-test of control group (n = 12) versus individual treatment groups (n = 12) for each miRNA.

In order to evaluate the effects of cytokines and growth factors on miRNA expression and exosomal-secretion in the liver, rat primary hepatocytes were stimulated for 24 hours with either FGF2, FGF4, HGF, IL-1α, IL-6, INF-β or TGF-β1. At the end of the treatment, cells and conditioned medium were collected and isolation of both total and exosomal RNAs were carried out as described. Following RNA quality control and cDNA synthesis, the expression of a panel of miRNAs was analyzed by qPCR (Fig. 7). Importantly, miRNAs included in this panel were selected with respect to their expression in the liver (miR-122^31^, miR-194^31^ and miR-192^31^), their function during infection and inflammation (miR-155^34^,^35^ and miR-150^36^,^37^), their pro-fibrotic activity (miR-142-3p^38^), or their altered expression during fibrosis or hepato-cellular carcinoma (miR-30d^39^, miR-98^40^, miR-92a^41^, miR-21^42^, miR-18a^43^ and miR-223^42^,^44^). Analysis of cellular miRNAs identified that administration of FGF2, FGF4 or INF-β significantly regulated the expression of several miRNAs (Fig. 7a) including the liver-enriched miR-194^31^. miR-194 expression is regulated by hepatocyte nuclear factor 1α (HNF1-α^45^) and its down-regulation may have an effect on cellular mobility^46^. Additionally, our data indicate that administration of FGF4 along with IL-1α and INF-β significantly down-regulates miR-21, which regulates cell cycle progression during mouse liver regeneration^47^. Overall, we observed that the expression of cellular miRNAs shows a trend toward the down-regulation, suggesting that the activity of extracellular signals on hepatocytes may decouple miRNA-mediated translational repression. On the other hand, levels of exosomal-miRNAs were mostly up-regulated by the treatments (Fig. 7b). Moreover, it can be observed that exosomal-miRNAs preferentially respond to cytokines administration, in contrast to cellular miRNAs that preferentially respond to growth factors. Specifically, IL-6 and TGF-β1 regulated the expression of 53% (6 out of the 11) and 63% (7 out of the 11) of the exosome-secreted miRNAs respectively. Changes in levels of exosomal-miR-98 were not analyzed since miR-98 was not detectable in exosomes secreted by control primary hepatocytes (data not shown). Overall, this data suggests a complex interplay between the signaling pathways down-stream to cytokines and growth factors in the modulation of miRNAs expression and exosomal-secretion, interaction that will be further investigate in future studies.

The presented data indicate that miQPCR greatly simplified the analysis of these experiments by significantly reducing the sample handling. For this study, four independent experiments were performed, where each independent experiment included triplicates for the eight different conditions. For each experiment 24 cellular and 24 exosomal RNAs were investigate, requiring the synthesis of 192 individual cDNAs to complete the whole analysis. If we had measured the expression of 12 different miRNAs using similar analysis performed with TaqMan miRNA-assays, which requires individual cDNAs to be synthesized through miRNA-specific stem-loop reverse transcription primers^32^, we would have required the synthesis of 2304 individual cDNAs.

## Discussion

In little more than a decade miRNAs have deeply affected every field of biology and medicine and their discovery has effectively modified the way we view and approach the regulation of gene expression as well as open up new possibility in the search of clinically relevant biomarkers^15^. Herewith, we described ‘miQPCR’ an innovative approach for the accurate and sensitive quantification of miRNAs by using qPCR. With the miQPCR method, we established a method for achieving the universal reverse transcription of all the miRNAs contained in the RNA sample. Essential components of this system are: i) the miLINKER, an oligonucleotide adapter encompassing the sequence required for ligation and reverse transcription; ii) the Rnl2tr, which joins the 5′-end of the miLINKER with the 3′-end of miRNAs; iii) the mQ-RT primer that reverse transcribes and extends the ligated miRNAs to their final size; and iv) the *Tm* adjusted miRNA specific primers. Importantly, T4 RNA ligases are a powerful class of enzymes enabling the application of several methods including miRNA cloning^48^,^49^, miRNA labeling for microarray^33^, miRNA labeling for liquid phase detection assays^50^, cDNA synthesis^28^ and in the generation of small RNA library for next generation sequencing^51^.

Although the miQPCR method has not been published before, we have previously shown that the miQPCR is a suitable technique for measuring miRNA level in RNAs extracted from tissues, primary cells and cell lines^52^, from serum^53^, as well as from RNA extracted from Formalin–fixed and paraffin–embedded (FFPE) specimens (11; and Supplementary Figure 2). Notably, beside miQPCR no other approaches enable the ‘universal’ or ‘multiplexed’ elongation and reverse transcription of miRNAs. In the first approach, *E. coli* Poly (A) Polymerase (PAP) is used to add, in a template independent fashion, adenosine nucleotides to the 3′-end of all the RNA contained in the sample. While in the latter approach TaqMan Low density miRNA Array card (TLDA, Applied Biosystem) enables the parallel screening of large number of miRNAs by using qPCR (PAP and TLDA have been reviewed in^54^). Drawback of these methods compared to miQPCR is that [unless Locked Nucleic Acid (LNA) are used] the PAP does not allow the design of miRNA-specific primers discriminating between mature and miRNA precursors, whereas TLDAs consist of custom-made 384-well micro fluidic cards, which are not flexible in their design. Furthermore, TLDAs requires the acquisition of dedicate equipment that will considerably increase the price for reaction.

The recent discovery of cell-free miRNAs circulating in the blood of healthy as well as diseased individuals^16^,^17^, have raised the possibility that significant changes in the levels of specific miRNAs may reflect the clinical manifestation of the disease^20^. Hence, to demonstrate the ability of miQPCR to expression profile miRNAs in both cellular and exosomal-isolated RNAs, we used miQPCR to measure the expression levels of cellular and exosomal-miRNAs in rat primary hepatocytes stimulated with a panel of cytokines and growth factors. Interestingly, we discovered that both cytokines and growth factors were able to affect the expression or the secretion of cellular or exosomal-miRNAs (Fig. 7a,b). Our data clearly show that expression of miRNAs in hepatocytes tends to be down-regulated by the treatments. Of note, miR-21, a well-characterized oncomiR regulating cell cycle progression^47^, is significantly down-regulated by FGF4, IL-1α and INF-β administration, suggesting a potential tumor-suppressor or anti-proliferative activity of these factors on hepatocytes. On the other hand, we observed that IL-6 and TGF-β1 administration up-regulated between 50 and 60% of the analyzed exosomal-miRNAs, suggesting a pleiotropic activity of these cytokines on the composition or secretion of exosomal-miRNAs. Importantly, we noticed that the up-regulation of a specific miRNAs in the exosomes is not associated with its down-regulation at cellular level, suggesting that cellular and exosomal-miRNAs are regulated via different signaling pathways. Future experiments will focus on the analysis and characterization of molecular pathways down- and up-stream to the regulated cellular miRNAs, as well as in assessing the functionality of exosomal-associated miRNAs in respect of inter-cellular communication.

In summary, we have created an innovative, simple, flexible, robust and inexpensive tool for monitoring the expression profile of miRNAs by using qPCR. The advantages of the miQPCR protocol over existing miRNA-qPCR platform are: i) a ‘one-step’ reverse transcription of all RNAs contained in the sample; ii) *Tm* adjustment of miRNA-specific primers; iii) simple and flexible primer design and; iv) an open and cost effective platform, which achieve optimal performance of the qPCR assay. Based on the presented data we expect that miQPCR can greatly facilitate miRNA expression profiling in biological and clinical samples.

## Methods

### miLINKER and mQ-RT design

The miQPCR method consists in two distinct steps, the RNA elongation and reverse transcription. RNA elongation is achieved through the ligation of the miLINKER (a 26 nts long linker-adaptor; IDT USA; Supplementary Table 1) to RNAs 3′-ends. For increased specificity, the miLINKER sequence was derived from the tuberosum phyB gene (GeneBank Y14572) which has been optimized not to hybridize with any known sequences in published genomes. miLINKER is designed to be a substrate of the truncated T4 RNA ligase 2 K227Q (Rnl2tr, NEB Cat: M0242L). For this purpose, the linker was synthesized with a 5′, 5′-adenyl group at the 5′-end, while a Dideoxycytidine group blocks the linker’s 3′-end (Fig. 1a and Supplementary Table 1a). The miLINKER ligation to mature miRNAs (22–24 nts) results in the formation of a molecule 48 to 50 nts long. The reverse transcription via an optimized reverse transcription primer named mQ-RT, which brings the final size of the amplicon to 59 to 61 nts that is the optimal amplicon range for qPCR. qPCR assays are run with a miRNA specific primer and a universal reverse primer named Upm2A (Fig. 1b).

### Rat primary hepatocyte preparation, cytokine stimulation and RNA isolation

All experiments and experimental protocols involving animals were approved by and conducted in accordance with the guidelines of the University of Düsseldorf Institutional Animal Care and Use Committee. Primary hepatocytes were isolated from male Wistar rats (150–200 gr) essentially as described^55^. In brief, hepatocytes were isolated after serial perfusion of rat liver by Hanks’s balanced salt solution (HBSS, Sigma Cat: H6648) and collagenase CLS type II solution (50 mg/150 ml, Biochrom Cat: C2-22). The collagenase was dissolved in HBSS (Sigma Cat: H8264) supplemented with albumin fraction V (3 gr/150 ml, Roth Cat: CP84.1) and applied by circulated perfusion for 17–20 minutes at 37 °C. After sufficient digestion, a pair of tweezers was used to disrupt the liver tissue and the resulting cell suspension was centrifuged three times at 44 × g for 3 minutes to remove non-parenchymal cells. The hepatocyte pellet was suspended in culture medium (DMEM-F12, Gibco Cat: 11320-074) supplemented with 10% (v/v) fetal calf serum (FCS, Biochrom Cat: S0615) and 1% (v/v) penicillin-streptomycin-amphotericin B solution (Gibco Cat: 15240-062). About 6.6*10^5^ hepatocytes per well were plated out using 6-well plates (Greiner Cat: 657169). The plastic surface of the 6-well plates was pre-coated by collagen type I (Sigma Cat: C3867, 6–10 μg/cm^2^). The adherent hepatocytes received new medium after 3 hours of culture at 37 °C and 5% CO_2_. Primary hepatocytes were allowed to recover for 24 hours and the medium was then switched to a serum-free medium comprising of DMEM-F12 (Gibco), 1% (v/v) penicillin-streptomycin-amphotericin B solution (Gibco) and 1% (v/v) linoleic acid/bovine serum albumin solution (Sigma Cat: L9530) for additional 24 hours. Subsequently, primary hepatocytes were stimulated with 1.5 ml of serum-free medium containing either cytokines or growth factors [TGF-β1 (10 ng/ml; Sigma Cat: T5050), IL-6 (50 ng/ml; Sigma Cat: I0406), IL-1α (25 ng/ml; Sigma Cat: I3901), INF-β (1000 U; Millipore Cat: IF011), HGF (20 ng/ml; PreproTech Cat: 100-39), FGF2 (100 ng/ml; Sigma Cat: F0291) and FGF4 (100 ng/ml; PreproTech Cat: 100-31)], or cultured in 1.5 ml serum free medium (control) for 24 hours.

### Isolation, purification of cellular and exosomal RNAs

Following treatment of primary hepatocytes with cytokines and growth factors, conditioned mediums were collected and stored frozen while cell were rinsed in cold PBS, scrapped from the wells and lysate in 500 μl of Qiazol. Cellular RNAs were isolated with the miRNeasy mini kit (Qiagen Cat: 217004) by following manufacturer instructions and eluted in 50 μl of nuclease free water. To isolate exosomal-miRNAs, conditioned mediums were thaw and pass through 0.45 μm filter syringe. Following, 500 μl of Total Exosome Isolation Reagent (Invitrogen Cat: 4478359) were added to 1 ml of filtered medium and incubated ON at 4 °C. Following incubations, the samples were centrifuged at 10000 × g for 60 minutes at 4 °C and the resulting exosomal pellets dissolved in 500 μl of Qiazol. Exosomal RNAs were isolated according to the miRNeasy protocol and eluted in 50 μl of nuclease free water. Concentrations and quality of cellular RNAs were estimated respectively by using NanoDrop ND-100 (Thermo Scientific) and non-denaturing agarose gel.

### Genome wide analysis of miRNAs with miCHIP

MiRNA expression profiling was performed using the miCHIP microarray platform as described^33^,^56^. In brief, 500 ng of FirstChoice liver or heart total RNA were labeled with a Cy3–conjugated RNA linker (Biospring, Frankfurt, Germany) and hybridized on the microarray. miCHIP is based on locked nucleic acid (LNA) technology, whereby LNA–modified Tm–normalized miRCURY capture probes (Exiqon, Denmark) based on miRBase release 11 were printed on Codelink slides (GE Healthcare, Munich, Germany). Microarray images were generated using the Genepix 4200AL laser scanner (Molecular Devices, Biberach and der Riss, Germany) in batches using the Genepix auto PMT (Photo Multiplayer). The ‘MultiExperiment Viewer’ MeV was used to execute the statistical technique SAM (Significance Analysis of Microarrays) to identify miRNAs of interest.

### cDNA synthesis and qPCR analysis following the miQPCR and the TaqMan platforms

#### miQPCR assay

The presented method exploits the characteristic of Rnl2tr (NEB; Cat M0242L) and PrimeScript (Takara; Cat 2680A) to respectively elongate and reverse transcriptase elongated miRNAs. To the purpose of elongate miRNAs, 10 ng of total RNA (or 4 μl of miRNeasy isolated Exosomal-RNAs) are dilute into 4 μl of nuclease free water, mixed with 4 μl of *Elongation Mix* (Table 1a) and incubated for 30 minutes at 25 °C. At the end of the incubation, samples are hold at 10 °C. Following, 7 μl of *cDNA Mix1* (Table 1b) were added to the ligated RNAs and incubated at 85 °C for 2 minutes followed by cooling to 46 °C. In the last step, 5 μl of *cDNA Mix2* (Table 1c) are then added to each sample (Final volume 20 μl) and elongate miRNAs are reverse transcribed at 46 °C for 30 minutes followed by 5 minutes at 85 °C. At the end of the reverse transcriptase inactivation samples are hold at 10 °C. cDNAs are diluted to 50 pg/μl by the addition of 180 μl of nuclease-free water (final volume 200 μl) and stored at −20 °C until use. For qPCR assays, 2 μl of diluted cDNA (equivalent to 100 pg) was mixed with primers, SYBR Green I (Life Technologies, Cat: 4367659) and nuclease free water (for the detailed qPCR master mix see Table 1d) and run on a 7500 Real-Time PCR instrument (Applied Biosystems). The 7500 cycler was programmed as follow: 95 °C for 10 minutes, followed by 50 cycles of 95 °C for 10 seconds, 60 °C for 35 seconds, including dissociation step (ramping from 60 °C to 95 °C) for monitoring melting curve of the amplification products. Calculation for the optimal miLINKER (Supplementary Figure 3) and Poly Ethylene Glycol (PEG; Supplementary Figure 4) concentrations are included in the Supplementary material and methods section.

#### TaqMan miRNA assay

cDNA for TaqMan assay were essentially prepared following the provider instructions. Briefly 10 ng of liver or heart total RNAs were reverse transcribed with individual stem-loop RT-primers for miR-1 (Cat: 002222), miR-16 (Cat: 000391), miR-133a (Cat: 002246), miR-122 (Cat: 000445), miR-192 (Cat: 000491), miR-194 (Cat: 000493), miR-21 (Cat: 000397) and U6 (Cat: 001973). Following reverse transcription, one (1) ng of each individually synthesized cDNA was used in the qPCR assay with TaqMan probes.

All the cDNAs syntheses were carried out in 200 ml PCR tubes in a PCR cycler (PCT-225 Thermal Cycler, MJ Researcher). Heart and liver total RNA used in comparison between the different platforms were purchased from Life Technologies (FirstChoice mouse total RNA, Life Technologies Cat: AM7816 and AM7810). Relative miRNAs expressions were determined by using the ΔΔCt methods^57^ within qBase^58^ or manually in Microsoft Excel.

### Synthetic miRNAs and miRNA standard curves

16.5 fmol (equivalent to 1*10^9^ copies) of synthetic miRNAs (Let-7a, Let-7b, Let-7c, Let-7d, Let-7e and Let-7f) were spiked into 50 ng of yeast RNA. cDNA was synthesized from 10 ng of spiked RNAs (containing 2*10^8^ copies) as described above. cDNAs were diluted with nuclease free water and the equivalent of 100 pg of reverse transcribed RNA (containing 2*10^6^ copies) were amplified by using Upm2A and each of the optimization primers designed to amplify the selected members of the Let-7 family (Supplementary Table 1c). Yeast total RNA was chosen to create a complex environment as it was shown that yeast RNA does not contains miRNAs-like molecules^59^.

For the determination of standard curves, 10 ng of liver total RNAs were reverse transcribed following the miQPCR protocol. Following reverse transcription, nuclease free water was used to bring the final volume of the cDNAs to 200 μl (or 50 pg/μl) and seven 1:5 linear dilutions were prepared (Fig. 5). Following, 2 μl of each dilution was analyzed in qPCR assays by using Upm2A universal primers and miR-122, miR-16, miR-192 or miR-21 as miRNA-specific primers (the sequences of the primers used in this study are listed in the Supplementary Table 1b and 1c).

### Prediction of duplex molecule *Tms*

Melting temperatures of individual sequences were calculated by using the freely available “*Tm* calculator” tool (see below). The *Tm* of the complete miRBase v19 represented in Fig. 2 and included in Supplementary Table 2 were calculated by using Breslauer thermodynamic parameters^60^.

### Statistical analysis and World Wide Web tools

To evaluate whether the observed differences between two groups were significant we employed the unpaired T-test. Statistical analyses were calculated either with Microsoft Excel or by using QuickCalcs t test calculator, GraphPad Software, Inc. Accessed 12 November 2014, (http://www.graphpad.com/quickcalcs/ttest1.cfm?Format=SD).

Sequence of mature miRNA was downloaded from the miRBase database^27^: (http://www.mirbase.org/).

The predicted *Tms* of the duplex molecule were calculated by using the web based *Tm* calculator: (http://www.appliedbiosystems.com/support/techtools/calc/).

Primers were analyzed for the presence of primer-dimer and secondary structure by using the web based OligoAnalyzer from IDT: (http://eu.idtdna.com/analyzer/Applications/OligoAnalyzer/Default.aspx).

## Additional Information

**How to cite this article**: Benes, V. *et al*. Identification of cytokine-induced modulation of microRNA expression and secretion as measured by a novel microRNA specific qPCR assay. *Sci. Rep*. **5**, 11590; doi: 10.1038/srep11590 (2015).

## Supplementary Material

Supplementary Information

Supplementary Table S2

## Figures and Tables

**Table 1:** Composition of the different master mixes required to carry out cDNA synthesis and qPCR analysis by using miQPCR.

miQPCR master mixes calculations (INCLUDING 10% extra volume in calculations):			
	Volume (μl)	X Samples	Check
a) Elongation Mix			
10X T4 Rnl2 Buffer (NEB)	0.90		□
150 mM MgCl2 (final 5 μM)	0.30		□
50% PEG 8000 (final 15%)	2.70		□
miLINKER (5 mM)	0.30		□
RNase Inhibitor (40 U/μl)	0.10		□
Rnl2tr K227Q (NEB)	0.20		□
b) cDNA Mix1			
dNTPS (10 mM)	0.50		□
ddH2O (Nuclease Free)	7.0		□
mQ-RT primer (10 mM)	0.25		□
c) cDNA Mix2			
5X RT Buffer (Takara)	4.40		□
PrimeScript (Takara)	0.25		□
ddH2O (Nuclease Free)	0.85		□
			
**qPCR master mix calculation (extra volume NOT included in calculation):**			
d) qPCR Master Mix			
ddH2O (Nuclease Free)	7.4		□
Upm2A primer (10 mM)	0.30		□
miRNA primer (10 mM)	0.30		□
2X SYBR Green I mix	10.0		□

## References

[b1] Lai E. C. Micro RNAs are complementary to 3′ UTR sequence motifs that mediate negative post-transcriptional regulation. Nat Genet 30, 363–364 (2002). 11896390 10.1038/ng865

[b2] Han J. . Molecular basis for the recognition of primary microRNAs by the Drosha-DGCR8 complex. Cell 125, 887–901 (2006). 16751099 10.1016/j.cell.2006.03.043

[b3] Ketting R. F. . Dicer functions in RNA interference and in synthesis of small RNA involved in developmental timing in C. elegans. Genes Dev 15, 2654–2659 (2001). 11641272 10.1101/gad.927801 PMC312808

[b4] Gregory R. I., Chendrimada T. P., Cooch N. & Shiekhattar R. Human RISC couples microRNA biogenesis and posttranscriptional gene silencing. Cell 123, 631–640 (2005). 16271387 10.1016/j.cell.2005.10.022

[b5] Zekri L., Huntzinger E., Heimstadt S. & Izaurralde E. The silencing domain of GW182 interacts with PABPC1 to promote translational repression and degradation of microRNA targets and is required for target release. Mol Cell Biol 29, 6220–6231, MCB.01081-09 (2009). 19797087 10.1128/MCB.01081-09 PMC2786699

[b6] Hornstein E. . The microRNA miR-196 acts upstream of Hoxb8 and Shh in limb development. Nature 438, 671–674 (2005). 16319892 10.1038/nature04138

[b7] Chen X. A microRNA as a translational repressor of APETALA2 in Arabidopsis flower development. Science 303, 2022–2025 (2004). 12893888 10.1126/science.1088060 PMC5127708

[b8] Sinkkonen L. . MicroRNAs control *de novo* DNA methylation through regulation of transcriptional repressors in mouse embryonic stem cells. Nat Struct Mol Biol 15, 259–267, nsmb.1391 (2008). 18311153 10.1038/nsmb.1391

[b9] Hatfield S. D. . Stem cell division is regulated by the microRNA pathway. Nature 435, 974–978 (2005). 15944714 10.1038/nature03816

[b10] Poy M. N. . A pancreatic islet-specific microRNA regulates insulin secretion. Nature 432, 226–230 (2004). 15538371 10.1038/nature03076

[b11] Castoldi M. . The liver-specific microRNA miR-122 controls systemic iron homeostasis in mice. J Clin Invest 121, 1386–1396, 10.1172/JCI44883 (2011). 21364282 PMC3069782

[b12] Lu J. . MicroRNA expression profiles classify human cancers. Nature 435, 834–838 (2005). 15944708 10.1038/nature03702

[b13] Ozen M., Creighton C. J., Ozdemir M. & Ittmann M. Widespread deregulation of microRNA expression in human prostate cancer. Oncogene 27, 1788–1793, 1210809 (2008). 17891175 10.1038/sj.onc.1210809

[b14] Dalmay T. & Edwards D. R. MicroRNAs and the hallmarks of cancer. Oncogene 25, 6170–6175 (2006). 17028596 10.1038/sj.onc.1209911

[b15] Jeffrey S. S. Cancer biomarker profiling with microRNAs. Nat Biotechnol 26, 400–401, 10.1038/nbt0408-400 (2008). 18392022

[b16] Weber J. A. . The microRNA spectrum in 12 body fluids. Clin Chem 56, 1733–1741, 10.1373/clinchem.2010.147405 (2010). 20847327 PMC4846276

[b17] McDonald M. K., Capasso K. E. & Ajit S. K. Purification and microRNA profiling of exosomes derived from blood and culture media. Journal of visualized experiments : JoVE, e50294, 10.3791/50294 (2013). 23792786 PMC3727427

[b18] Arroyo J. D. . Argonaute2 complexes carry a population of circulating microRNAs independent of vesicles in human plasma. Proc Natl Acad Sci U S A 108, 5003–5008, 10.1073/pnas.1019055108 (2011). 21383194 PMC3064324

[b19] Ludwig A. K. & Giebel B. Exosomes: small vesicles participating in intercellular communication. Int J Biochem Cell Biol 44, 11–15, 10.1016/j.biocel.2011.10.005 (2012). 22024155

[b20] Schwarzenbach H., Nishida N., Calin G. A. & Pantel K. Clinical relevance of circulating cell-free microRNAs in cancer. Nature reviews. Clinical oncology 11, 145–156, 10.1038/nrclinonc.2014.5 (2014). 24492836

[b21] Sugino A., Snoper T. J. & Cozzarelli N. R. Bacteriophage T4 RNA ligase. Reaction intermediates and interaction of substrates. J Biol Chem 252, 1732–1738 (1977). 320212

[b22] Viollet S., Fuchs R. T., Munafo D. B., Zhuang F. & Robb G. B. T4 RNA ligase 2 truncated active site mutants: improved tools for RNA analysis. BMC Biotechnol 11, 72, 10.1186/1472-6750-11-72 (2011). 21722378 PMC3149579

[b23] Lee K. S. . Functional role of NF-kappaB in expression of human endothelial nitric oxide synthase. Biochem Biophys Res Commun 448, 101–107, 10.1016/j.bbrc.2014.04.079 (2014). 24769202

[b24] Ando Y. . Overexpression of microRNA-21 is associated with elevated pro-inflammatory cytokines in dominant-negative TGF-beta receptor type II mouse. J Autoimmun 41, 111–119, 10.1016/j.jaut.2012.12.013 (2013). 23395552 PMC3622842

[b25] Kim J. S. . MicroRNA-205 directly regulates the tumor suppressor, interleukin-24, in human KB oral cancer cells. Mol Cells 35, 17–24, 10.1007/s10059-013-2154-7 (2013). 23212344 PMC3887855

[b26] Szabo G. & Bala S. MicroRNAs in liver disease. Nature reviews. Gastroenterology & hepatology 10, 542–552, 10.1038/nrgastro.2013.87 (2013). PMC4091636 23689081

[b27] Griffiths-Jones S., Saini H. K., van Dongen S. & Enright A. J. miRBase: tools for microRNA genomics. Nucleic Acids Res 36, D154–158, gkm952 (2008). 17991681 10.1093/nar/gkm952 PMC2238936

[b28] Munafo D. B. & Robb G. B. Optimization of enzymatic reaction conditions for generating representative pools of cDNA from small RNA. RNA 16, 2537–2552, 10.1261/rna.2242610 (2010). PMC2995414 20921270

[b29] Heyer A. & Gatz C. Isolation and characterization of a cDNA-clone coding for potato type B phytochrome. Plant Mol Biol 20, 589–600 (1992). 1450376 10.1007/BF00046444

[b30] Nolan T., Hands R. E., Ogunkolade W. & Bustin S. A. SPUD: a quantitative PCR assay for the detection of inhibitors in nucleic acid preparations. Anal Biochem 351, 308–310, 10.1016/j.ab.2006.01.051 (2006). 16524557

[b31] Landgraf P. . A mammalian microRNA expression atlas based on small RNA library sequencing. Cell 129, 1401–1414 (2007). 17604727 10.1016/j.cell.2007.04.040 PMC2681231

[b32] Chen C. . Real-time quantification of microRNAs by stem-loop RT-PCR. Nucleic Acids Res 33, e179 (2005). 16314309 10.1093/nar/gni178 PMC1292995

[b33] Castoldi M., Schmidt S., Benes V., Hentze M. W. & Muckenthaler M. U. miChip: an array-based method for microRNA expression profiling using locked nucleic acid capture probes. Nat Protoc 3, 321–329, nprot.2008.4 (2008). 18274534 10.1038/nprot.2008.4

[b34] Yin Q., Wang X., McBride J., Fewell C. & Flemington E. K. B-cell receptor activation induces BIC/MIR-155 expression through a conserved AP-1 element. J Biol Chem 283, 2654–62 (2008). 18048365 10.1074/jbc.M708218200 PMC2810639

[b35] Tili E. . Modulation of miR-155 and miR-125b Levels following Lipopolysaccharide/TNF-{alpha} Stimulation and Their Possible Roles in Regulating the Response to Endotoxin Shock. J Immunol 179, 5082–5089 (2007). 17911593 10.4049/jimmunol.179.8.5082

[b36] Zhou B., Wang S., Mayr C., Bartel D. P. & Lodish H. F. miR-150, a microRNA expressed in mature B and T cells, blocks early B cell development when expressed prematurely. Proc Natl Acad Sci U S A 104, 7080–7085 (2007). 17438277 10.1073/pnas.0702409104 PMC1855395

[b37] Xiao C. . MiR-150 Controls B Cell Differentiation by Targeting the Transcription Factor c-Myb. Cell 131, 146–159 (2007). 17923094 10.1016/j.cell.2007.07.021

[b38] Zhu H., Luo H. & Zuo X. MicroRNAs: their involvement in fibrosis pathogenesis and use as diagnostic biomarkers in scleroderma. Exp Mol Med 45, e41, 10.1038/emm.2013.71 (2013). 24052166 PMC3789263

[b39] Yao J. . MicroRNA-30d promotes tumor invasion and metastasis by targeting Galphai2 in hepatocellular carcinoma. Hepatology 51, 846–856, 10.1002/hep.23443 (2010). 20054866

[b40] Hou W., Tian Q., Steuerwald N. M., Schrum L. W. & Bonkovsky H. L. The let-7 microRNA enhances heme oxygenase-1 by suppressing Bach1 and attenuates oxidant injury in human hepatocytes. Biochim Biophys Acta 1819, 1113–1122, 10.1016/j.bbagrm.2012.06.001 (2012). 22698995 PMC3480558

[b41] Tan W., Li Y., Lim S. G. & Tan T. M. miR-106b-25/miR-17-92 clusters: polycistrons with oncogenic roles in hepatocellular carcinoma. World J Gastroenterol 20, 5962–5972, 10.3748/wjg.v20.i20.5962 (2014). 24876719 PMC4033436

[b42] Karakatsanis A. . Expression of microRNAs, miR-21, miR-31, miR-122, miR-145, miR-146a, miR-200c, miR-221, miR-222, and miR-223 in patients with hepatocellular carcinoma or intrahepatic cholangiocarcinoma and its prognostic significance. Mol Carcinog 52, 297–303, 10.1002/mc.21864 (2013). 22213236

[b43] Vettori S., Gay S. & Distler O. Role of MicroRNAs in Fibrosis. The open rheumatology journal 6, 130–139, 10.2174/1874312901206010130 (2012). 22802911 PMC3396185

[b44] Xu J. . Circulating microRNAs, miR-21, miR-122, and miR-223, in patients with hepatocellular carcinoma or chronic hepatitis. Mol Carcinog 50, 136–142, 10.1002/mc.20712 (2011). 21229610

[b45] Krutzfeldt J. . MicroRNA-194 is a target of transcription factor 1 (Tcf1, HNF1alpha) in adult liver and controls expression of frizzled-6. Hepatology 55, 98–107, 10.1002/hep.24658 (2012). 21887698

[b46] Meng Z. . miR-194 is a marker of hepatic epithelial cells and suppresses metastasis of liver cancer cells in mice. Hepatology 52, 2148–2157, 10.1002/hep.23915 (2010). 20979124 PMC3076553

[b47] Ng R., Song G., Roll G. R., Frandsen N. M. & Willenbring H. A microRNA-21 surge facilitates rapid cyclin D1 translation and cell cycle progression in mouse liver regeneration. J Clin Invest 122, 1097–1108, 10.1172/JCI46039 (2012). 22326957 PMC3287214

[b48] Pfeffer S., Lagos-Quintana M. & Tuschl T. Cloning of small RNA molecules. Curr Protoc Mol Biol Chapter 26, Unit 26 24, 10.1002/0471142727.mb2604s72 (2005). 18265364

[b49] Ambros V. & Lee R. C. Identification of microRNAs and other tiny noncoding RNAs by cDNA cloning. Methods Mol Biol 265, 131–158 (2004). 15103073 10.1385/1-59259-775-0:131

[b50] Neely L. A. . A single-molecule method for the quantitation of microRNA gene expression. Nat Methods 3, 41–46 (2006). 16369552 10.1038/nmeth825

[b51] Morin R. D. . Application of massively parallel sequencing to microRNA profiling and discovery in human embryonic stem cells. Genome Res, gr.7179508 (2008). 10.1101/gr.7179508 PMC2279248 18285502

[b52] Ibberson D., Benes V., Muckenthaler M. U. & Castoldi M. RNA degradation compromises the reliability of microRNA expression profiling. BMC Biotechnol 9, 102, 1472-6750-9-102 (2009). 20025722 10.1186/1472-6750-9-102 PMC2805631

[b53] Hentzschel F. . AAV8-Mediated *In Vivo* Overexpression of miR-155 Enhances the Protective Capacity of Genetically Attenuated Malarial Parasites. Mol Ther, 10.1038/mt.2014.172 (2014). PMC4429697 25189739

[b54] Benes V. & Castoldi M. Expression profiling of microRNA using real-time quantitative PCR, how to use it and what is available. Methods, S1046-2023(10)00041-1 (2010). 10.1016/j.ymeth.2010.01.026 20109550

[b55] Berry M. N. & Friend D. S. High-yield preparation of isolated rat liver parenchymal cells: a biochemical and fine structural study. J Cell Biol 43, 506–520 (1969). 4900611 10.1083/jcb.43.3.506 PMC2107801

[b56] Castoldi M. . A sensitive array for microRNA expression profiling (miChip) based on locked nucleic acids (LNA). Rna 12, 913–920 (2006). 16540696 10.1261/rna.2332406 PMC1440900

[b57] Livak K. J. & Schmittgen T. D. Analysis of relative gene expression data using real-time quantitative PCR and the 2(-Delta Delta C(T)) Method. Methods 25, 402–408 (2001). 11846609 10.1006/meth.2001.1262

[b58] Hellemans J., Mortier G., De Paepe A., Speleman F. & Vandesompele J. qBase relative quantification framework and software for management and automated analysis of real-time quantitative PCR data. Genome Biol 8, R19, gb-2007-8-2-r19 (2007). 17291332 10.1186/gb-2007-8-2-r19 PMC1852402

[b59] Lim L. P., Glasner M. E., Yekta S., Burge C. B. & Bartel D. P. Vertebrate microRNA genes. Science 299, 1540 (2003). 12624257 10.1126/science.1080372

[b60] Breslauer K. J., Frank R., Blocker H. & Marky L. A. Predicting DNA duplex stability from the base sequence. Proc Natl Acad Sci U S A 83, 3746–3750 (1986). 3459152 10.1073/pnas.83.11.3746 PMC323600

