# Supplementary material for: Identification of cytokine-induced modulation of microRNA expression and secretion as measured by a novel microRNA specific qPCR assay
Source: Sci Rep. 2015 Jun 25;5:11590. doi: 10.1038/srep11590 (PMC4480321; doi:10.1038/srep11590)
Supplement: Supplementary Information [file srep11590-s1.pdf]

Identification of cytokine-induced modulation of microRNA expression and secretion as measured by a novel microRNA specific qPCR assay

Vladimir Benes, Paul Collier, Claus Kordes, Jens Stolte, Tobias Rausch, Martina U. Muckentaler, Dieter Häussinger and Mirco Castoldi

A **Microarray analysis of miRNA expression in mouse liver and heart**

**Median normalized values**								
**Heart**	**Rep-01**	**Rep-02**	**Rep-03**	**Rep-04**	**Rep-05**	**Rep-06**	**Average**	**SD**
**miR-1**	161.43	140.75	170.10	163.75	192.86	136.08	**160.83**	**20.67**
**miR-122**	0.50	0.33	0.43	0.44	0.36	0.41	**0.41**	**0.06**
**miR-133b**	47.80	44.52	46.60	55.15	60.68	39.41	**49.03**	**7.66**
**miR-16**	6.77	7.59	5.96	6.48	7.05	6.79	**6.77**	**0.55**
**miR-192**	0.57	0.67	0.47	0.43	0.57	0.48	**0.53**	**0.09**
**miR-194**	1.84	2.81	1.58	1.78	2.29	1.96	**2.04**	**0.44**
**miR-21**	2.78	3.17	2.92	2.76	2.49	2.98	**2.85**	**0.23**

**Liver**	**Rep-01**	**Rep-02**	**Rep-03**	**Rep-04**	**Rep-05**	**Rep-06**	**Average**	**SD**
**miR-1**	40.82	55.94	39.14	46.98	48.83	44.20	**45.99**	**6.08**
**miR-122**	118.69	214.65	148.51	109.65	262.25	197.45	**175.20**	**59.72**
**miR-133b**	NA	0.33	0.15	0.23	0.24	0.17	**0.22**	**0.07**
**miR-16**	0.83	1.14	1.37	0.71	0.88	0.51	**0.90**	**0.31**
**miR-192**	1.66	1.67	1.20	1.47	1.27	1.77	**1.51**	**0.23**
**miR-194**	3.07	3.04	3.02	2.59	2.39	2.97	**2.85**	**0.29**
**miR-21**	1.56	1.64	1.08	1.41	0.97	1.71	**1.40**	**0.31**

B **TaqMan (imput material 1 ng/qPCR run)**

**Gene**	**Tissue**	**Ct Average**	**SD**	**Tissue**	**Ct Average**	**SD**	**ΔΔCt**	**Liver**	**Heart**	**Even**
**miR-1**	Heart	17.00	0.22	LIVER	24.59	0.17	192.57			
**miR-122**	Heart	32.60	0.24	LIVER	20.49	0.11	4436.33			
**miR-133b**	Heart	15.47	0.08	LIVER	23.49	0.11	257.89			
**miR-16**	Heart	18.15	0.14	LIVER	20.59	0.06	5.42			
**miR-192**	Heart	25.45	0.06	LIVER	19.75	0.13	51.94			
**miR-194**	Heart	25.99	0.03	LIVER	22.07	0.11	15.11			
**miR-21**	Heart	20.14	0.15	LIVER	20.19	0.34	1.04			
**RNU6**	Heart	20.11	0.27	LIVER	20.06	0.52	0.96			

miQPCR (imput material 100 pg/qPCR run)

**Gene**	**Tissue**	**Ct Average**	**SD**		**Ct Average**	**SD**	**ΔΔCt**	**Liver**	**Heart**	**Even**
**miR-1**	Heart	19.82	0.44	LIVER	26.63	0.23	112.24			
**miR-122**	Heart	29.52	0.12	LIVER	18.31	0.57	2382.34			
**miR-133b**	Heart	21.52	0.14	LIVER	26.79	0.70	38.65			
**miR-16**	Heart	19.69	0.06	LIVER	21.89	0.20	4.59			
**miR-192**	Heart	26.90	0.42	LIVER	22.73	0.70	18.00			
**miR-194**	Heart	26.11	0.05	LIVER	22.62	0.55	11.25			
**miR-21**	Heart	22.32	0.05	LIVER	22.18	0.38	0.91			
**RNU6**	Heart	19.80	0.17	LIVER	19.45	0.12	0.78			

Supplementary Figure 1

Identification of cytokine-induced modulation of microRNA expression and secretion as measured by a novel microRNA specific qPCR assay

Vladimir Benes, Paul Collier, Claus Kordes, Jens Stolte, Tobias Rausch, Martina U. Muckentaler, Dieter Häussinger and Mirco Castoldi

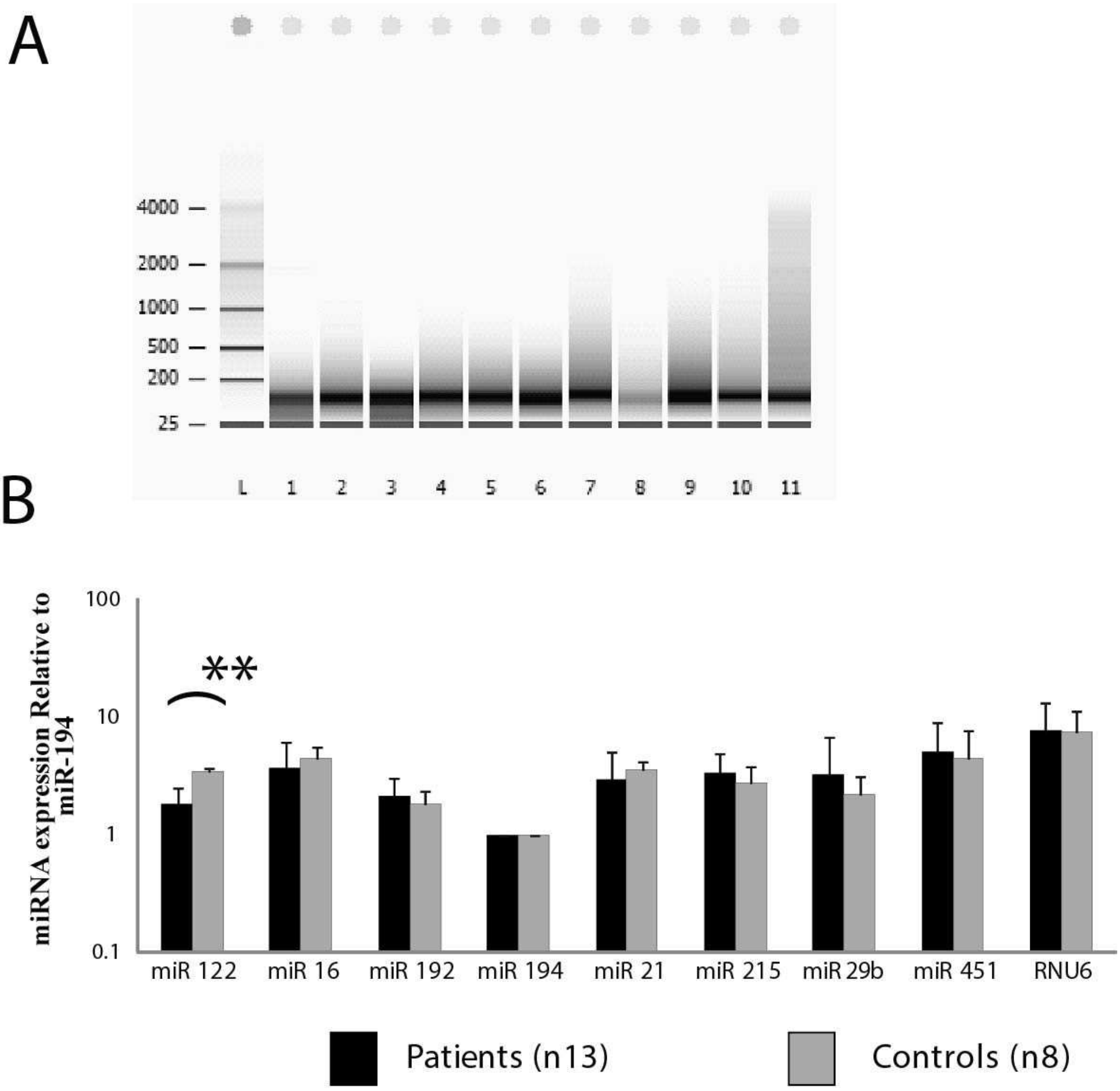

Supplementary Figure 2

Identification of cytokine-induced modulation of microRNA expression and secretion as measured by a novel microRNA specific qPCR assay

Vladimir Benes, Paul Collier, Claus Kordes, Jens Stolte, Tobias Rausch, Martina U. Muckentaler, Dieter Häussinger and Mirco Castoldi

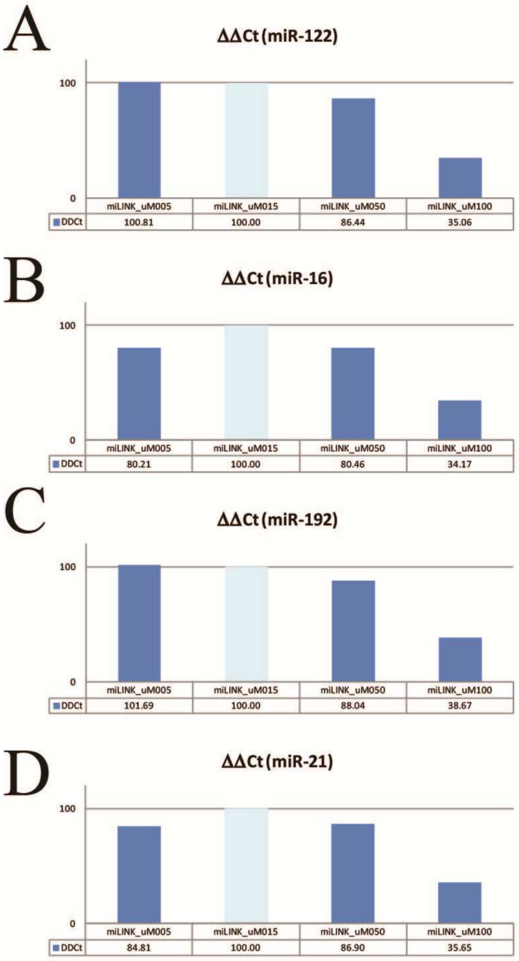

E

miLINKER	Target Name	Average	SD	ΔCt	ΔΔCt
miLINK_uM005	miR-122	20.90	0.26	0.01	100.81
miLINK_uM015	miR-122	20.91	0.94	0.00	100.00
miLINK_uM050	miR-122	21.12	0.49	-0.21	86.44
miLINK_uM100	miR-122	22.42	0.19	-1.51	35.06
NegRT_005	miR-122				
NegRT_015	miR-122				
NegRT_050	miR-122	45.39			
NegRT_100	miR-122	48.36		ΔCt	ΔΔCt
miLINK_uM005	miR-16	22.97	0.15	-0.32	80.21
miLINK_uM015	miR-16	22.66	0.53	0.00	100.00
miLINK_uM050	miR-16	22.97	0.22	-0.31	80.46
miLINK_uM100	miR-16	24.21	0.09	-1.55	34.17
NegRT_005	miR-16	46.68			
NegRT_015	miR-16	41.80			
NegRT_050	miR-16	44.23			
NegRT_100	miR-16	39.87		ΔCt	ΔΔCt
miLINK_uM005	miR-192	26.05	0.28	0.02	101.69
miLINK_uM015	miR-192	26.08	0.83	0.00	100.00
miLINK_uM050	miR-192	26.26	0.47	-0.18	88.04
miLINK_uM100	miR-192	27.45	0.30	-1.37	38.67
NegRT_005	miR-192				
NegRT_015	miR-192				
NegRT_050	miR-192	35.88			
NegRT_100	miR-192	33.84		ΔCt	ΔΔCt
miLINK_uM005	miR-21	23.29	0.32	-0.24	84.81
miLINK_uM015	miR-21	23.06	0.91	0.00	100.00
miLINK_uM050	miR-21	23.26	0.41	-0.20	86.90
miLINK_uM100	miR-21	24.54	0.16	-1.49	35.65
NegRT_005	miR-21				
NegRT_015	miR-21				
NegRT_050	miR-21				
NegRT_100	miR-21				

Supplementary Figure 3

Identification of cytokine-induced modulation of microRNA expression and secretion as measured by a novel microRNA specific qPCR assay

Vladimir Benes, Paul Collier, Claus Kordes, Jens Stolte, Tobias Rausch, Martina U. Muckentaler, Dieter Häussinger and Mirco Castoldi

A

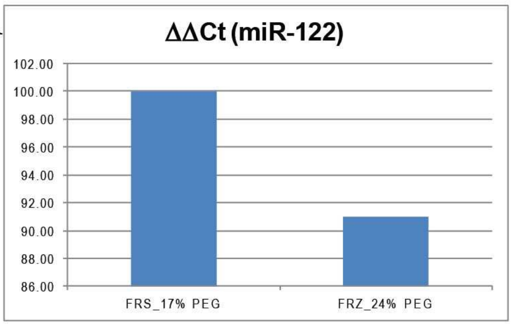

D

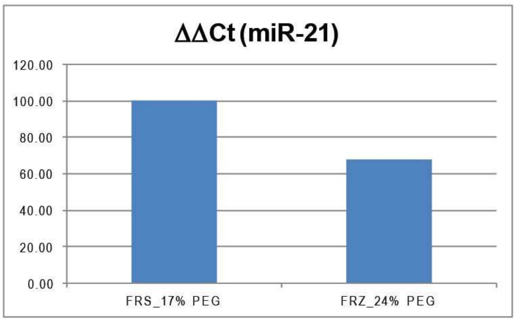

B

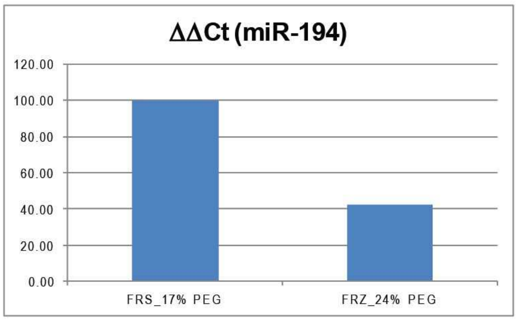

E

Sample Name	Target Name	Average	SD	ΔCt	ΔΔCt
FRS_17% PEG	miR-122	20.37	0.14	0.00	100.00
FRZ_24% PEG	miR-122	20.51	0.41	-0.14	90.97
FRS_17% PEG	miR-16	22.28	0.11	0.00	100.00
FRZ_24% PEG	miR-16	23.08	0.42	-0.80	57.40
FRS_17% PEG	miR-194	24.95	0.29	0.00	100.00
FRZ_24% PEG	miR-194	26.19	0.48	-1.24	42.48
FRS_17% PEG	miR-21	21.82	0.14	0.00	100.00
FRZ_24% PEG	miR-21	22.38	0.47	-0.56	67.88

C

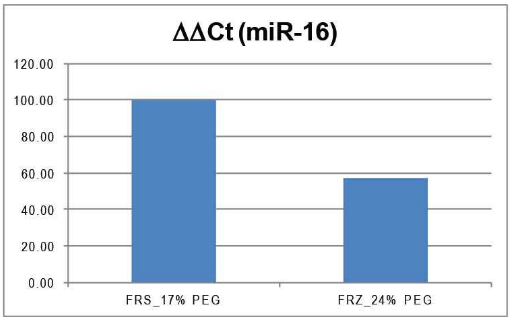

Supplementary Figure 4

Identification of cytokine-induced modulation of microRNA expression and secretion as measured by a novel microRNA specific qPCR assay

Vladimir Benes, Paul Collier, Claus Kordes, Jens Stolte, Tobias Rausch, Martina U. Muckentaler, Dieter Häussinger and Mirco Castoldi

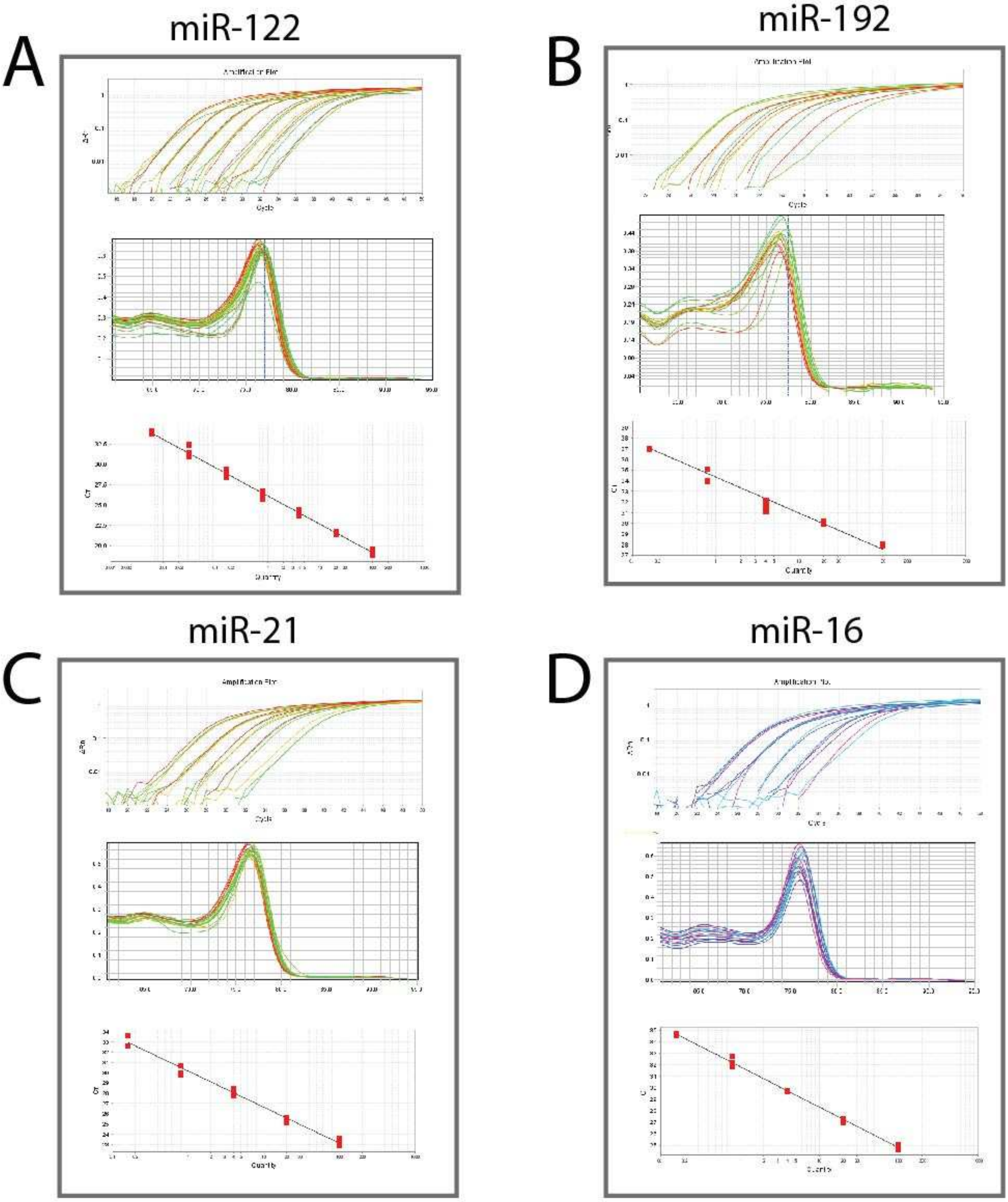

Supplementary Figure 5

Identification of cytokine-induced modulation of microRNA expression and secretion as measured by a novel microRNA specific qPCR assay

Vladimir Benes, Paul Collier, Claus Kordes, Jens Stolte, Tobias Rausch, Martina U. Muckentaler, Dieter Häussinger and Mirco Castoldi

Supplementary Table 1

A

NAME	Sequences 5' > 3'
miLINKER	pp(r)A.GGCCGAACTACGACCTGCATAAACGG.ddC
mQ-RT	CCCAGTTATGGCCGTTTATGCAGGT

B

NAME	Sequences 5' > 3'
Upm2A	CCCAGTTATGGCCGTTTA
miR-1	TGGAATGTAAAGAAGTATGTATGG
miR-122	GAGTGTGACAATGGTGTTTGG
miR-133b	GGTCCCCTTCAACCAGCTAG
miR-142-3p	TGTAGTGTTTCCTACTTTATGGAG
miR-150	CCAACCCTTGTACCAGTGG
miR-155	TTAATGCTAATTGTGATAGGGGTG
miR-16	CAGCACGTAAATATTGGCGG
miR-18a	GGTGCATCTAGTGCAGATAGG
miR-192	TGACCTATGAATTGACAGCCG
miR-194	GTAACAGCAACTCCATGTGGAG
miR-21	TAGCTTATCAGACTGATGTTGAGG
miR-223	TCAGTTTGTCAAATACCCCAG
miR-30d	TCCCCGACTGGAAGG
miR-92a	CTTGTCCCGGCCTGG
miR-98	AGGTAGTAAGTTGTATTGTTGG
RNU6	GCAAGGATGACACGCAAATT

C

NAME	Sequences 5' > 3'
rno-miR-122-5p	GAGTGTGACAATGGTGTTTGG
rno-miR-122-5p(-G)	AGTGTGACAATGGTGTTTG
rno-miR-122-3p	CGCCATCATCACACTAAG
rno-miR-122-3p(-G)	AACGCCATCATCACACTAA
rno-miR-21-5p	GCTTATCAGACTGATGTTGAG
rno-miR-21-5p(-G)	GCTTATCAGACTGATGTTGA
let-7a-5p	AGTAAGGTTGTATAGTTG
let-7b-5p	TAGTAGGTTGTGTGGTTG
let-7c-5p	TAGTAGGTTGTATGGTTG
let-7d-5p	AGAGGTAGTAGGTTGCATAGTTG
let-7e-5p	TAGGAGGTTGTATAGTTG
let-7f-5p	AGTAGATTGTATAGTTGG

Identification of cytokine-induced modulation of microRNA expression and secretion as measured by a novel microRNA specific qPCR assay

Vladimir Benes, Paul Collier, Claus Kordes, Jens Stolte, Tobias Rausch, Martina U. Muckentaler, Dieter Häussinger and Mirco Castoldi

Supplementary Figure Legends

Supplementary Figure 1: Detection, analysis and comparison of miRNAs expression in mouse liver and heart by using miQPCR and TaqMan miRNA assays. A) Microarray analysis of miRNA expression in FirstChoice total RNA from mouse heart or liver. Following hybridization, array data were median normalized and the average of six independent experiments was calculated. Data are indicated as mean ± SD (n=6). **B**) cDNA was synthesized using miQPCR from FirstChoice total RNA extracted mouse liver or heart. Expression profiling of miR-1, miR-122, miR-133b, miR-16, miR-192, miR-194, miR-21 and RNU6 was determined using miQPCR or TaqMan miRNA assays. Data are indicated as mean ± SD (n=4). Four independent experiments were performed and the ΔΔCt of heart vs liver were calculated. Data with higher expression in the liver is represented in blue, in the heart is represented in red and invariant signal is represented in green.

Supplementary Figure 2: miQPCR analysis of miRNA expression in RNA extracted from human FFPE biopsies. A) Assessment of RNA integrity. RNA extracted from human FFPE biopsies was analyzed on an Agilent Pico Chip (Bioanaylzer). The presence of low molecular weight smears indicates complete RNAs degradation. **B**) miQPCR analysis of miRNAs expression in RNA extracted from FFPE liver biopsies of patients and control groups. Only the expression levels of miR-122 are significantly reduced in the liver of patients compared to control group. Data are represented as mean ± SD (patient n=13; controls n=8). To determine whether the differences between two groups were significant we employed the unpaired T-test, $**p < 0.01$.

Identification of cytokine-induced modulation of microRNA expression and secretion as measured by a novel microRNA specific qPCR assay

Vladimir Benes, Paul Collier, Claus Kordes, Jens Stolte, Tobias Rausch, Martina U. Muckentaler, Dieter Häussinger and Mirco Castoldi

Supplementary Figure 3: Optimization of the miQPCR elongation protocol, identification of the miLINKER working concentrations. RNAs were elongate and reverse transcribed by using standard miQPCR approach by including different the amount of miLINKER used in the elongation step (0.5, 1.5, 5 and 10 μM), measurement for different miRNAs **A**) miR-122, **B**) miR-16, **C**) miR-192 and **D**) miR-21. **E**) Indicates the average and standard deviation calculated from four independent experiments. ΔCts and ΔΔCts were calculated relative to measures performed with cDNAs synthesized with 1.5 μM of miLINKER. Average and standard deviation from four (n=4) independent experiments are shown.

Supplementary Figure 4: Calibration of the miQPCR elongation protocol, identification of the PEG working concentrations. RNAs were elongate and reverse transcribed by using standard miQPCR approach by including two different amounts of PEG (17% and 24%), measurement for different miRNAs **A**) miR-122, **B)** miR-194, **C**) miR-16 and **D**) miR-21. **E**) Indicates the average and standard deviation calculated from six independent experiments. ΔCts and ΔΔCts were calculated relative to measures performed with cDNAs synthesized with 17% PEG. Average and standard deviation from six (n=6) independent measurements are shown.

Supplementary Figure 5: Amplification plots, melting curves and standard curves of hepatic miRNAs. 10 ng of liver total RNA (FirstChoice) were reverse transcribed using the miQPCR protocol and 1:5 scalar dilutions prepared (100 pg, 20 pg, 4 pg, 800 fg, 160 fg, 32 fg and 6 fg) and the expression of 4 miRNAs **A**); miR-122, **B**); miR-192, **C**); miR-21 and **D**)

Identification of cytokine-induced modulation of microRNA expression and secretion as measured by a novel microRNA specific qPCR assay

Vladimir Benes, Paul Collier, Claus Kordes, Jens Stolte, Tobias Rausch, Martina U. Muckentaler, Dieter Häussinger and Mirco Castoldi

miR-16) was analyzed by qPCR as described. Data are represented as Ct average ± standard deviation calculated from four (n=4) independent cDNAs synthesis.

Supplementary Table 1: Primers used in this study. A) linker-adaptor and RT primers used for miQPCR cDNA synthesis and **B and C**) miQPCR primers used in the qPCR assays included in this study.

Supplementary Table 2: Complete list of *Tm* adjusted primers included in the miRBase v19. The sequences for all miRNAs were downloaded from miRBase (version 19) and their *Tms* were calculated before (*Tm*) and after (*Tm*2) adjustment. Predicted *Tm* for all the sequences were calculated by using Breslauer thermodynamic parameters.

Identification of cytokine-induced modulation of microRNA expression and secretion as measured by a novel microRNA specific qPCR assay

Vladimir Benes, Paul Collier, Claus Kordes, Jens Stolte, Tobias Rausch, Martina U. Muckentaler, Dieter Häussinger and Mirco Castoldi

SUPPLEMENTARY MATERIALS AND METHODS

Design of miQPCR miRNA-specific primers

The specificity and sensitivity of qPCR assays rely on primer design. For the intrinsic nature of the mature miRNA, design of miRNA-specific primers is particularly challenging. An important aspect of the miQPCR approach is that miLINKER addition creates an amplicon of around 60 nts (it vary according to the initial miRNA-length), which consents for the flexible design of miRNA-specific primer allowing the achievement of a consistent *Tm*. To design miRNA-specific primers follow the stepwise optimization of primer design for miQPCR:

a. Download the full-length sequence of the mature miRNAs from the miRBase database, as starting point the design for *Tm* adjusted primers of all miRNAs contained in miRBase v19 is available in the supplementary Supplemental Table 2.

b. Add a G to the 3'-end of the miRNA sequence, which is the first nucleotide of the miLINKER sequence (Supplemental Table 1).

c. Determine the predicted *Tm* of the duplex molecule (see web links at the end of On-line Material and Methods)

d. If the predicted *Tm* is 55°C> *Tm*< 62°C, then the sequence is suitable to be used without further modification.

e. If the predicted *Tm* is lower than 55°C, then elongate the 3'-end of the miRNA-specific primer sequence by further adding up to two additional nucleotides from the miLINKER 5'-end (Supplemental Table 1).

f. If the predicted *Tm* is higher than 62°C, then shorten the 5'-end of the miRNA sequence successively until a *Tm* between 55°Cand 62°C is reached.

g. As the shortening of the sequence may result in a loss of specificity, we strongly recommend blasting the obtained primer sequence against the miRBase: (http://www.mirbase.org/search.shtml)

Parameters critical for the success of the miQPCR

1) RNA quality control: to obtain accurate and reproducible qPCR data it is essential to use total RNA with an RNA Integrity Number (RIN) above 7 [1,2].

Identification of cytokine-induced modulation of microRNA expression and secretion as measured by a novel microRNA specific qPCR assay

Vladimir Benes, Paul Collier, Claus Kordes, Jens Stolte, Tobias Rausch, Martina U. Muckentaler, Dieter Häussinger and Mirco Castoldi

2) Starting amount of total RNA: experimental conditions have been optimized for of small RNAs contained within 10 ng of total RNA; for exosomal miRNAs extracted from Serum or tissue culture medium uses four µl (see RNA extraction for detailed RNA isolation protocol).

3) miLINKER sequence features: To allow the ligation by the truncated T4-RNA ligase 2 the linker adaptor must contain one adenyl group at its 5'-end to ensure efficient ligation by the RNA ligase.

4) Amount of miLINKER: For 10 ng of total RNA (or 4 µl of exosomal RNA) the optimal final concentration of miLINKER is between 0.5 and 1.5 µM (data not shown). In the current protocol, we employ 1.5 µM of miLINKER.

5) Reverse transcription must be carried out at 46ºC. During cDNA synthesis, it is important to avoid cooling the mix below 46ºC following the denaturation step.

6) The presented data were generated with the Power SYBR Green master Mix (Life Technologies, Cat: 4367659) and PrimeScript (Takara-Clontech, Cat: 2680A) as reverse transcriptase. In addition to the reagent described in the manuscript, we have tested with positive results the following vendors for SYBR Green: Clontech (Cat: 638322 and Cat: 638321), Takara (Cat: RR820W), Promega (Cat: A6002) and Bioline (Cat: QT650-05) as well as the following reverse transcriptases: Superscript II (Invitrogen, Cat: 18064-014) and Superscript III (Invitrogen, Cat: 18080-044). T4 RNA ligases from other vendors were also tested, though none of the tried enzymes did offer results comparable to the Rnl2tr.

7) miQPCR have been successfully run on the following cyclers: ABI-ViiA7, ABI-7500, ABI-7900, ABI-StepOne Plus, Roche-Light Cycler, Illumina ECO, Analytik Jena qTOWER 2 and Fluidigm-BioMark-HD.

8) Cycler program is composed of a two-step qPCR with incubation at 95ºC for 10 minutes hot start to activate the polymerase (**IMPORTANT: the length of the hot-start step may differs among SYBR Green I sold by diverse vendors**), 50 cycles at 95ºC for 10 seconds and 60ºC for 35 seconds, followed by the melting curve.

Identification of cytokine-induced modulation of microRNA expression and secretion as measured by a novel microRNA specific qPCR assay

Vladimir Benes, Paul Collier, Claus Kordes, Jens Stolte, Tobias Rausch, Martina U. Muckentaler, Dieter Häussinger and Mirco Castoldi

9) The presented miQPCR protocol is not compatible for fast cycling chemistry. We have tested with negative results fast cycling chemistry on the ABI-StepOne Plus, ABI-7500 Fast and Fluidigm-BioMark-HD.

References

1 Becker, C., Hammerle-Fickinger, A., Riedmaier, I. & Pfaffl, M. W. mRNA and microRNA quality control for RT-qPCR analysis. *Methods,* doi:S1046-2023(10)00025-3 (2010).

2 Ibberson, D., Benes, V., Muckenthaler, M. U. & Castoldi, M. RNA degradation compromises the reliability of microRNA expression profiling. *BMC Biotechnol* **9**, 102, doi:1472-6750-9-102 (2009)
